# *De novo* Genome Assembly of the Fungal Plant Pathogen *Pyrenophora semeniperda*

**DOI:** 10.1371/journal.pone.0087045

**Published:** 2014-01-27

**Authors:** Marcus M. Soliai, Susan E. Meyer, Joshua A. Udall, David E. Elzinga, Russell A. Hermansen, Paul M. Bodily, Aaron A. Hart, Craig E. Coleman

**Affiliations:** 1 Plant and Wildlife Sciences, Brigham Young University, Provo, Utah, United States of America; 2 USDA Forest Service, Rocky Mountain Research Station, Shrub Sciences Laboratory, Provo, Utah, United States of America; Imperial College Faculty of Medicine, United Kingdom

## Abstract

*Pyrenophora semeniperda* (anamorph *Drechslera campulata*) is a necrotrophic fungal seed pathogen that has a wide host range within the Poaceae. One of its hosts is cheatgrass (*Bromus tectorum*), a species exotic to the United States that has invaded natural ecosystems of the Intermountain West. As a natural pathogen of cheatgrass, *P. semeniperda* has potential as a biocontrol agent due to its effectiveness at killing seeds within the seed bank; however, few genetic resources exist for the fungus. Here, the genome of *P. semeniperda* isolate assembled from sequence reads of 454 pyrosequencing is presented. The total assembly is 32.5 Mb and includes 11,453 gene models encoding putative proteins larger than 24 amino acids. The models represent a variety of putative genes that are involved in pathogenic pathways typically found in necrotrophic fungi. In addition, extensive rearrangements, including inter- and intrachromosomal rearrangements, were found when the *P. semeniperda* genome was compared to *P. tritici-repentis*, a related fungal species.

## Introduction

The ascomycete fungal genus *Pyrenophora* (anamorph *Drechslera*) is comprised of graminicolous species often associated with leaf-spotting disease in crops and turf grasses [1]. The genus includes the agronomically-important species *P. teres*, *P. graminea*, *P. tritici-repentis*, which are responsible for barley net blotch, barley stripe and wheat tan spot diseases, respectively [2]–[4]. These *Pyrenophora* species are necrotrophic pathogens and the diseases they cause result in substantial crop losses each year. In contrast to these foliar pathogens, *P. semeniperda* is primarily a seed pathogen, although leaf spotting has also been reported in plants infected by this fungus [5].

One of the better-characterized *Pyrenophora* species is *P. tritici-repentis*, an economically important pathogen of wheat [6]. This ascomycete (anamorph *Drechslera tritici-repentis*) causes tan spot and chlorosis in its host and is responsible for grain losses averaging 5 to 15% but reaching up to 50% in conditions favoring disease development [7]–[9]. *Pyrenophora tritici-repentis* infects the leaves of its host using exotoxins that induce necrotic spotting surrounded by chlorotic zones. Manning et al. [10] recently reported the genome sequence of three isolates of *P. tritici-repentis* using whole-genome Sanger sequencing. The genome annotation yielded over 11,000 genes, which serves as a useful model and reference for the sequencing and annotation of other *Pyrenophora* genomes. The haploid nuclear genome of the sequenced *P. tritici-repentis* isolate contains eleven chromosomes with an estimated size of 37 Mb.

*Pyrenophora semeniperda* (anamorph *Drechslera campulata*) is also a generalist pathogen on a wide range of grass genera. The host range of *P. semeniperda* was first described by Wallace in 1959 [11]. Currently, it is believed to infect over 36 genera of annual and perennial grasses [12]. It has been reported to infect developing seeds under experimental conditions. This infection does not have any effect on seed maturation but effectively reduces subsequent seed germination and emergence of its hosts [13]–[15]. Under natural conditions in the field the pathogen primarily attacks mature seeds in the seed bank [14]. Black stromata protruding out of dead seeds are characteristic of infection by the fungus.

Interest has been expressed in using *P. semeniperda* as a biocontrol agent against cheatgrass (*Bromus tectorum*) [16]–[18], an invasive weed in the Intermountain West (IMW) of the United States. Cheatgrass is a threat to many ecosystems of the IMW, invading sensitive habitats of native plants and animals, and providing fuel for disastrous wildfires. As a natural pathogen of cheatgrass, *P. semeniperda* is effective at killing seeds after conidial inoculation [16] and its use as a biocontrol agent may offer a superior alternative to expensive and dangerous conventional methods of control such as herbicides or early season burning. Despite recent interest in the fungus and its potential as a biocontrol agent for cheatgrass, there are very few genetic and genomic resources available to facilitate studies of *P. semeniperda* biology.

Here, the *de novo* assembly of the *P. semeniperda* genome from 454 pyrosequencing reads and its annotation are presented. The small genome size, haploid state, and modest level of repetitive elements within many fungal genomes make the job of *de novo* assembly relatively simple compared to other larger and more complex eukaryotic genomes [19]. The *P. semeniperda* sequencing project has four main objectives: 1) Obtain a high-quality draft of the *P. semeniperda* genome using next-generation sequencing technology, 2) annotate the genome using *P. semeniperda* ESTs and sequencing data from *P. tritici-repentis* and other fungal genomes to validate gene models, 3) identify genes involved in pathogenicity, and 4) establish sequence co-linearity and orthology between *P. semeniperda* and *P. tritici-repentis* by identifying genomic structural variations. These objectives will help to elucidate factors involved in virulence and other molecular mechanisms that may be used to exploit the fungus to control expansion of cheatgrass populations. Moreover, the work presented here may add to the general knowledge of fungal biology and contribute to the discovery of novel mechanisms of pathogenicity and infection by other fungi.

## Materials and Methods

### DNA and RNA Isolation

Fungal cultures and tissue were prepared as described by Boose et al. [20]. A single *P. semeniperda* isolate (CCB06) was prepared from a *B. tectorum* seed bank sample collected at Cinder Cone Butte, Idaho, USA. The seed bank sample was obtained as part of a cooperative study with the Idaho Army National Guard, which has administrative responsibility for the Orchard Training Area where Cinder Cone Butte is located. DNA was isolated from mycelium using the ZR Fungal/Bacterial DNA MiniPrep™ kit (Zymo Research Corporation, Orange, CA) following the manufacturer's protocol. DNA was quantified using the NanoDrop ND-1000 spectrophotometer (NanoDrop products, Wilmington, DE).

RNA was isolated from two *P. semeniperda* isolates using the ZR Fungal/Bacterial RNA MiniPrep™ (Zymo Research Corporation, Orange, CA) and stored at −80 C; RNA was collected from multiple tissue types including mycelium, fruiting structures, and conidia from *P. semeniperda* isolates including the Cinder Cone Butte isolate used for genome sequence and an isolate collected from Skull Valley, Utah, USA. RNA quality and integrity was assessed for each extraction using the RNA Nano 6000 kit and the 2100 Bioanalyzer Expert software (Agilent Technologies, Santa Clara, CA). RNA quantity was measured with the TBS-380 Mini-Fluorometer (Turner Biosystems, Sunnyvale, CA) in combination with the RiboGreen RNA quantitation reagent (Molecular Probes, Eugene, OR).

### cDNA Library Construction and Normalization

RNA samples meeting sufficient quality and quantity criteria were pooled together for 1st strand synthesis and cDNA optimization. cDNA was synthesized from pooled RNA using the SMARTer™ PCR cDNA Synthesis Kit (ClonTech, Mountain View, CA) followed by PCR cycling of cDNA with the Advantage HF 2 PCR kit (ClonTech, Mountain View, CA). Modified oligo primers were used to allow for *MmeI* digestion for 5′ and 3′ adaptor excision: 5′ Smart Oligo [5′-AAG CAG TGG TAA CAA CGC ATC CGA CGC rGrGrG-3′]; 3′ Oligo dT SmartIIA [5′-AAG CAG TGG TAA CAA CGC ATC CGA CTT TTT TTT TTT TTT TTT TTT TTV N-3′]; New SmartIIA [5′-AAG CAG TGG TAA CAA CGC ATC CGA C-3′] (Sandra Clifton, Personal Communication, Washington University).

Normalization of cDNA was accomplished with the Axxora Trimmer cDNA Normalization kit (AxxOra, San Diego, CA). *MmeI* (New England BioLabs, Ipswitch, MA) was used for 5′ and 3′ modified SMART adaptor excision followed by removal of excised 5′ and 3′ adaptor ends using AMPure beads (Agencourt Bioscience Corporation, Beverly, MA) using manufacturer's recommended protocols.

### 454 Sequencing

In total, a half plate of a whole genome library, a full plate of a 3-kb paired end library, and a half plate of normalized cDNA were sequenced using the 454 Life Sciences Genome Sequencer using FLX Titanium series reagents (454 Life Sciences, Bradford, CT). Titanium emPCR, library preparation, and sequencing were completed at the Brigham Young University DNA Sequencing Center (Provo, Utah, USA).

### 454 Reads Assembly and Genome Annotation

*De novo* genome assembly was accomplished using all of the whole-genome shotgun and 3-kb paired-end reads with the Newbler software package (454 Life Sciences, Bradford, CT). Default settings were chosen for the assembly in Newbler. cDNA reads were assembled separately from genomic reads and default settings were chosen for transcript assembly.

The genome annotation pipeline MAKER [21] was used to predict gene models within the *de novo* assembly of *P. semeniperda*. Expressed sequence tags (ESTs), derived from the cDNA library, were used to provide evidence for predicted genes within the *P. semeniperda* genome for the annotation pipeline. An in-house Perl script was created to expedite the naming process, as an automated naming scheme did not exist within the MAKER pipeline.

*P. semeniperda* gene models were imported into the Blast2GO suite [22], [23] for functional annotation analysis. GO annotations were made in accordance with the recommended protocol in the Blast2Go tutorial. Default settings were chosen along with an e-value threshold set at ≤ e-06 for each step of the GO annotation process.

Repeated and low complexity sequences within the *P. semeniperda* genome were identified using RepeatMasker [24] with a fungal repeat library. A slow search was performed for increased accuracy, increasing the sensitivity of the search between 0–5%.

### Genome Assembly Validation

To assess the accuracy of the genome assembly, an automated validation program called amosvalidate was used to highlight regions of the genome that are suspected to be misassembled [25]. The amosvalidate pipeline returns features for each contig and scaffold that are likely to be errors in the assembly such as expansions or contractions of the reads that make up the assembly. Contig assemblies were imported into amosvalidate along with coordinates of the paired-end sequences for analysis. Hawkeye was used to visualize data from amosvalidate [26]. The amosvalidate output data was imported into Hawkeye where each scaffold was visually inspected for assembly errors.

### SyMap

SyMap v3.3 (Synteny Mapping and Analysis Program) [27] was used to generate dotplot displays of syntenic relationships between *P. semeniperda* and *P. tritici-repentis*. SyMap, by default uses NUCmer [28] for multiple genome alignments via a modified Smith-Waterman algorithm [29]. Gene descriptive information and other features associated with the *P. semeniperda* genome were imported into SyMap as GFFs (General Feature Files) after the alignment was completed.

## Results and Discussion

### Sequencing and assembly of the *P. semeniperda* genome

For sequencing, DNA was extracted from an isolate of *P. semeniperda* collected at Cinder Cone Butte, ID, USA. *Pyrenophora semeniperda* is haploid with an unknown chromosome number, although, electrophoretic and cytological karyotyping in related species reveals 9 chromosomes in *P. teres* [30] and from 8 to 11 chromosomes in *P. tritici-repentis*, depending on the isolate [31]. A shotgun strategy was used with the 454 Life Sciences Genome Sequencer FLX platform including whole-genome and 3 kb paired-end sequencing libraries. 454 Sequencing of the whole-genome shotgun (WGS) library on a half plate produced approximately 257 Mb of sequence with reads averaging 371 bp. The 3-kb paired-end library was sequenced on a full plate and produced over 469.9 Mb of sequence with an average read length of 362.06 bp. In total, 726.9 Mb of sequence was produced from 2,759,755 reads with an average read length of 366.7 bp; 28.11% (775,958) of the total were recognized as paired-end reads by the assembler and consequently used in the genome assembly (Table 1).

**Table 1:** Primary alignment metrics.

	Genomic Library	Paired Library	cDNA Library
no. of reads	691,955	2,067,802	333,014
no. of bases	233,183,002	469,930,696	110,891,692
avg. read length	371.47	362.06	358.2

The DNA sequence reads were assembled using the Newbler software package developed by 454 Life Sciences for *de novo* DNA sequence assembly. An incremental assembly approach was used where WGS reads were first used to create contigs based on an overlap, layout and consensus algorithm. The WGS reads were assembled into 7,890 contigs (N50  = 6,499); 98.38% of the total bases were successfully assembled into contigs (252 Mb). The initial assembly yielded a 6-fold coverage of the genome with approximately 31 Mb of aligned sequence. Next, reads from the 3-kb paired-end library were added to the assembly to provide read linkages that would span most repeats in the genome and increase the number of bases available for additional overlap and consensus. The inclusion of paired-end reads reduced the number of gaps from the assembly of the whole-genome reads alone (Table 2). The completed assembly project included 98.62% (672 Mb) of the total bases (681 Mb) and 95.00% of the raw reads (2,621,753 reads) assembled in 1,001 contigs (N50  = 104,587). The final coverage is 17X with the 1,001 contigs arranged into 54 scaffolds (N50  = 1.47 Mb); the 19 largest scaffolds represent 86.5% of the assembled *P. semeniperda* genome. The average distance between paired ends is 2.6 kb with a standard deviation of 665.5 bp. The estimated genome size of 40.1 Mb for *P. semeniperda* is similar to the reported size of 37.8 Mb for *P. tritici-repentis* [10] and 41.9 Mb for *P. teres* f. *teres* [30]. The 454 sequencing reads used in the assembly reported here are deposited in the NCBI sequence read archive (Accession: SRP007005). The whole genome shotgun project has been deposited at DDBJ/EMBL/GenBank under the accession ATLS00000000. The version described in this paper is version ATLS01000000.

**Table 2:** Progression of *P. semeniperda* Newbler assembly.

	Shotgun	Paired-end
**Peak Depth**	6	17
**Est. Genome Size**	42.6	40.1
**Read Status:**		
Num. Aligned Reads	678259	2621753
Num Aligned Bases	252629934	672385374
**Scaffold Metrics:**		
Number of Scaffolds		54
Number of Bases		32539172
Avg Scaffold Size		602577
N50* Scaffold Size		1473690
Largest Scaffold Size		2679305
**Large Contig Metrics:**		
Number of Contigs	7325	7777
Number of Bases	31201036	32316274
Avg Contig Size	4259	41591
N50 Contig Size	6499	104587
Largest Contig Size	42958	871975
**All Contig Metrics:**		
Number of Contigs	7890	1001
Number of Bases	31367229	32370938

* The N50 length describes 50% of the bases in the assembly that are in a contig/scaffold of at least that length.

### Genome Assembly Validation

The genome assembly was validated and visualized with Amosvalidate and Hawkeye respectively [25], [26]. Scaffolds and contigs were sorted from highest to lowest feature density and analyzed for major mis-assemblies in Hawkeye. With the exception of scaffold 30 the Compression-Expansion (CE) statistic, for the majority of scaffolds remains close to 0 and within the defaulted interval of −3 and +3, which indicates the likelihood of proper reads and mate-pair assembly. Clustering of mate-pair reads including compression and expansion of mate-pair reads is an indication of obvious mis-assembly and was not prevalent in the assembly. Validation results indicate 9 possible inversions in 9 different scaffolds and 1 possible insertion.

Clustering of expanded mate-pairs is observed throughout scaffold 30; expanded mate-pair clustering is normally an indication of insertion mis-assembly. Further investigation of the scaffold sequence show large amounts of homopolymers (mononucleotide repeats). Ambiguities and sequencing errors associated with long stretches of homopolymers are known to occur in 454 sequencing and may be the reason for expanded mate-pair clustering [32]. Scaffold 30 is relatively small (186 kb) and does not contain any annotated genes.

### Gene identification and annotation

The MAKER pipeline [21] was used to annotate the *P. semeniperda* genome and to create a publicly accessible genome database. The pipeline was used to make gene predictions, align ESTs to the genome, and integrate the ESTs into protein-coding gene annotations. To provide evidence of gene identity and ease of detection, a normalized *P. semeniperda* cDNA library was prepared for 454 sequencing. The library produced over 110.8 Mb of raw sequence data with read lengths averaging approximately 331 bp. The Newbler assembly of the cDNA sequence library generated 7,963 isogroups and over 7 Mb of total sequence length. In addition to EST evidence from *P. semeniperda*, 12,171 *P. tritici-repentis* gene models were used as a reference to reinforce confidence of *ab initio* gene predictions [10]. Each gene model was used as a query in a BLAST search against all protein sequences in the GenBank database, using an e-value cutoff threshold of hit scores ≤1×10^−20^.

**Figure 1:**
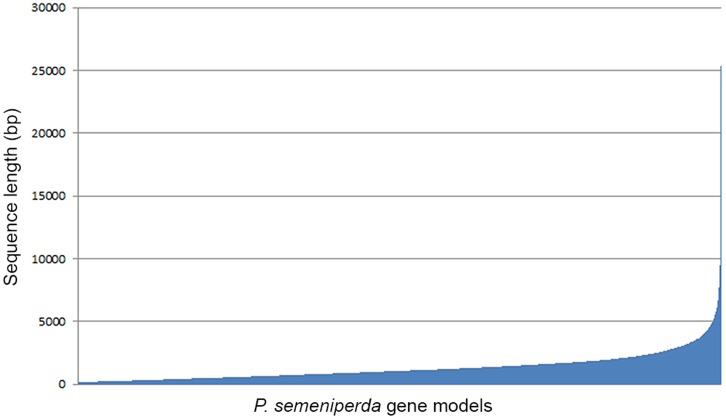
Length distribution of putative genes from *P. semeniperda*. Each of the 11,453 gene model is included on the X-axis from smallest to largest (left to right) with its length plotted on the Y-axis.

The MAKER pipeline predicted a total of 11,453 *ab initio* gene models, of which 9,578 yielded BLASTx hits to genes from other fungal species. Most of the top BLASTx hits were either to genes from *P. teres* (4,793) or *P. tritici-repentis* (3,995). Other fungal species for which there were top BLASTx hits included *Phaeosphaeria nodorum* (214) and *Leptosphaeria maculans* (173). No other fungal species provided more than 20 top BLASTx hits. The average coding sequence (CDS) length is 1,312 bp, ranging in size from 72 bp to 25,382 bp (Figure 1). The longest gene model is for a hypothetical protein that matched a gene model from *P. teres* in the BLASTx search.

### General genomic features

The overall GC content of the assembled *P. semeniperda* genome (32.029 Mb excluding gaps of unknown nucleotide sequence) is 49.98%. The GC content increases to 52.53% in gene coding sequences, which represent 46.47% of the genome. Analysis of the assembly using t-RNAscan-SE [33] detected 91 tRNA genes located on 22 scaffolds (Table 3). These putative tRNA genes do not group together as seen in other fungi such as *Saccharomyces pombe* [34]. Over 3,979 orthologous groups were identified between *P. semeniperda* and *P. tritici-repentis* with the Inparanoid v4.0 program [35], which describes genes derived from a common ancestor of the two fungal species; such genes are likely to share molecular function [36]. Also, 4,184 genes in *P. semeniperda* have been identified as in-paralogs (a result of gene duplication after a speciation event).

**Table 3:** General features of the *P. semeniperda* genome.

GC percentage in coding region	52.53
GC percentage in non-coding regions	47.73
tRNA genes	91
protein coding genes (CDSs)	11,453
percent coding	46.47
avg. CDS size (min/max)	1,312 bp (72 bp/25,382 bp)

### Gene ontology

**Figure 2:**
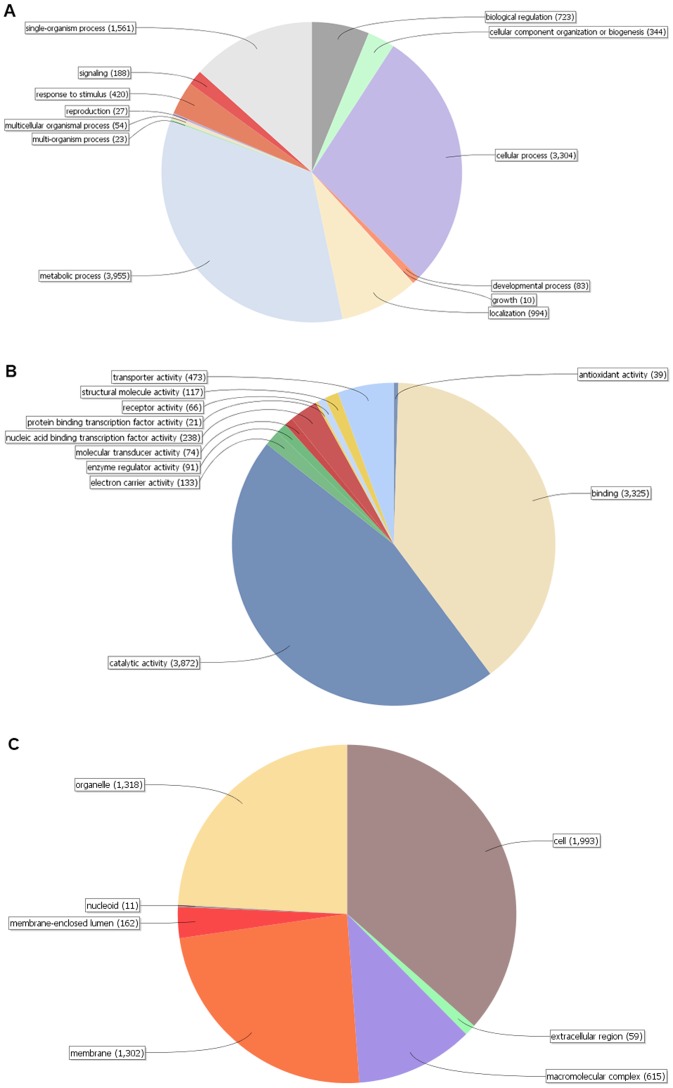
Distribution of Blast2GO annotations of putative genes from *P. semeniperda*. The charts show level 2 annotations for (A) Biological Process, (B) Molecular Function and (C) Cellular Components.

The set of 11,453 *P. semeniperda* gene models were analyzed using Blast2GO [22], [23] to identify gene function. From the query set, 6,419 genes were successfully annotated, yielding 25,595 GO terms. GO terms for the annotated genes were placed into three broad categories: biological process (BP), molecular function (MF), and cellular components (CC). Figures 2A, 2B and 2C are pie charts showing the distribution of GO terms at Level 2 for the three categories. The most abundant GO terms in the BP category (11,686 total terms) were metabolic process (34%), cellular process (28%), single-organism process (13%) and localization (9%) (Figure 2A). Abundant GO terms in the MF category (8,449 total terms) include catalytic activity (46%), binding (39%), and transporter activity (6%) (Figure 2B). Finally, most of the CC terms (5,460 total terms) are categorized as cell (37%), organelle (24%), membrane (24%) or macromolecular complex (11%) (Figure 2C). These GO terms only describe putative genes within the *P. semeniperda* genome and does not document gene expression.

### Repeat sequences and transposons

Transposable elements and repeated sequences are some of the most abundant sequences in eukaryotic genomes; for example, over 44% of the human genome [37] and more than 75% of the maize genome [38] are comprised of transposable and other repetitive elements. Fungal genomes, however, contain relatively small amounts of these elements when compared to other eukaryotes, rarely exceeding 5% of the genome [39]. Low levels of transposable and repetitive elements in fungal genomes may be due to defense mechanisms known as repeat-induced point mutations (RIP) [40] that protect fungal genomes against highly repeated sequences. The *P. semeniperda* genome was analyzed for repetitive sequences and retro-elements using RepeatMasker 3.2.7 [24], which screens DNA sequences for interspersed repeats and low complexity elements. Interspersed repeats (retroelements and DNA transposons) were the most abundant elements identified by RepeatMasker, totaling 610.7 kb or 1.89% of the genome. There were 447 class I retroelements identified, 388 of which were Gypsy/DIRS1 LTRs. Also found were 296 class II DNA transposable elements, 293 of which are Tc1-IS630-Pogo DNA transposons. In total, 859,266 bp or 2.66% of the genome was identified as containing interspersed repeats or low complexity elements (Table 4). This percentage is quantitatively consistent with the frequency of repeat elements observed in other ascomycete fungi, which rarely exceeds 5% of the genome [39].

**Table 4:** Identified interspersed and simple repeats in *P. semeniperda*.

	number of elements	length occupied (bp)	% of genome sequence
**Retroelements**	447	362220	1.12%
LINEs:	8	2295	0.01%
R1/LOA/Jockey	1	79	0%
LTR elements:	439	359925	1.11%
Ty1/Copia	51	38056	0.12%
Gypsy/DIRS1	388	321869	1%
**DNA transposons**	296	248288	0.77%
hobo-Activator	1	40	0%
Tc1-IS630-Pogo	293	248200	0.77%
MuDR-IS905	1	41	0%
**Unclassified:**	2	192	0%
**Total interspersed repeats:**		610700	1.89%
**Small RNA:**	47	12191	0.04%
**Simple repeats:**	2988	138005	0.43%
**Low complexity:**	1521	98370	0.30%

### Genome rearrangements

Questions have been raised concerning the impact transposable and repetitive elements have on the genomic architecture and evolution of fungi [39]. The presence of transposable elements in a genome can impact the regulation of neighboring genes and may provide sites for homologous and ectopic recombination [41]–[43]. Recombination sites may play an important role in observed local or wide-scale chromosomal rearrangements in fungi as well as in other organisms [44]–[48]. To investigate the role transposable and repeat elements may have played in the genomic architecture of *P. semeniperda*, its genome assembly was aligned with that of *P. tritici-repentis*. The *P. tritici-repentis* genome was ideal for a whole genome alignment as many of its sequences are arranged into full chromosomes, thereby allowing easier identification of genomic rearrangements within contigs or scaffolds of the *P. semeniperda* genome sequence.

**Figure 3:**
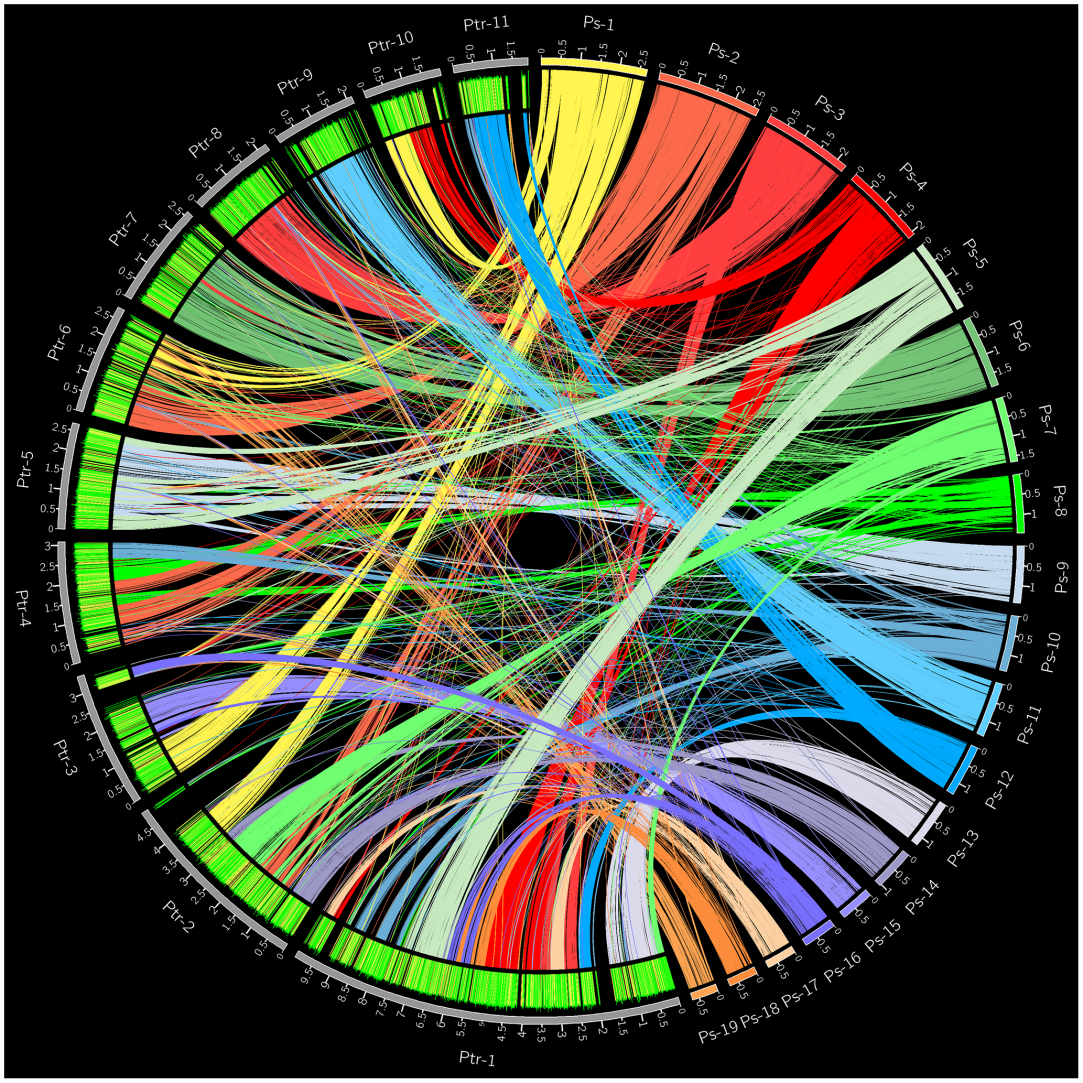
Circular view of the genome alignment between 19 *P. semeniperda* (Ps) scaffolds and 11 *P. tritici-repentis* (Ptr) chromosomes. The numbers marked on each scaffold or chromosome indicate length in megabases.

NUCmer [49] and Circos [50] was used to align and visualize genomic synteny between *P. semeniperda* and *P. tritici-repentis* (Figure 3). The 19 largest *P. semeniperda* scaffolds, representing 86.5% of the sequenced genome of *P. semeniperda*, were aligned to the 11 *P. tritici-repentis* chromosomes. The alignment produced 88% and 80% of syntenic coverage in *P. semeniperda* and *P. tritici-repentis*, respectively, and 6,376 gene hits on *P. semeniperda* scaffolds. Over 8,070 genes within the *P. semeniperda* assembly were aligned to the *P. tritici-repentis* genome, despite only including 19 of the 54 scaffolds in the genome alignment.

**Figure 4:**
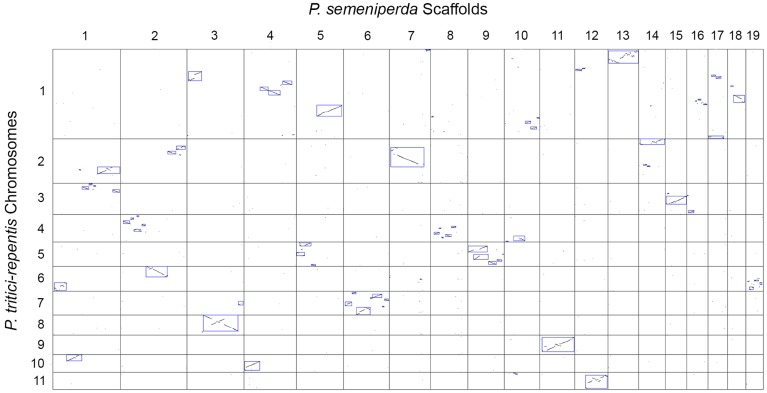
SyMap dot plot of genome alignment between *P. semeniperda* scaffolds and *P. tritici-repentis* chromosomes. The *P. semeniperda* scaffolds are numbered along the x-axis across the top and the *P. tritici-repentis* chromosomes are numbered on the y-axis along the left of the figure. Boxes highlight regions of homology between the two genomes.

**Figure 5:**
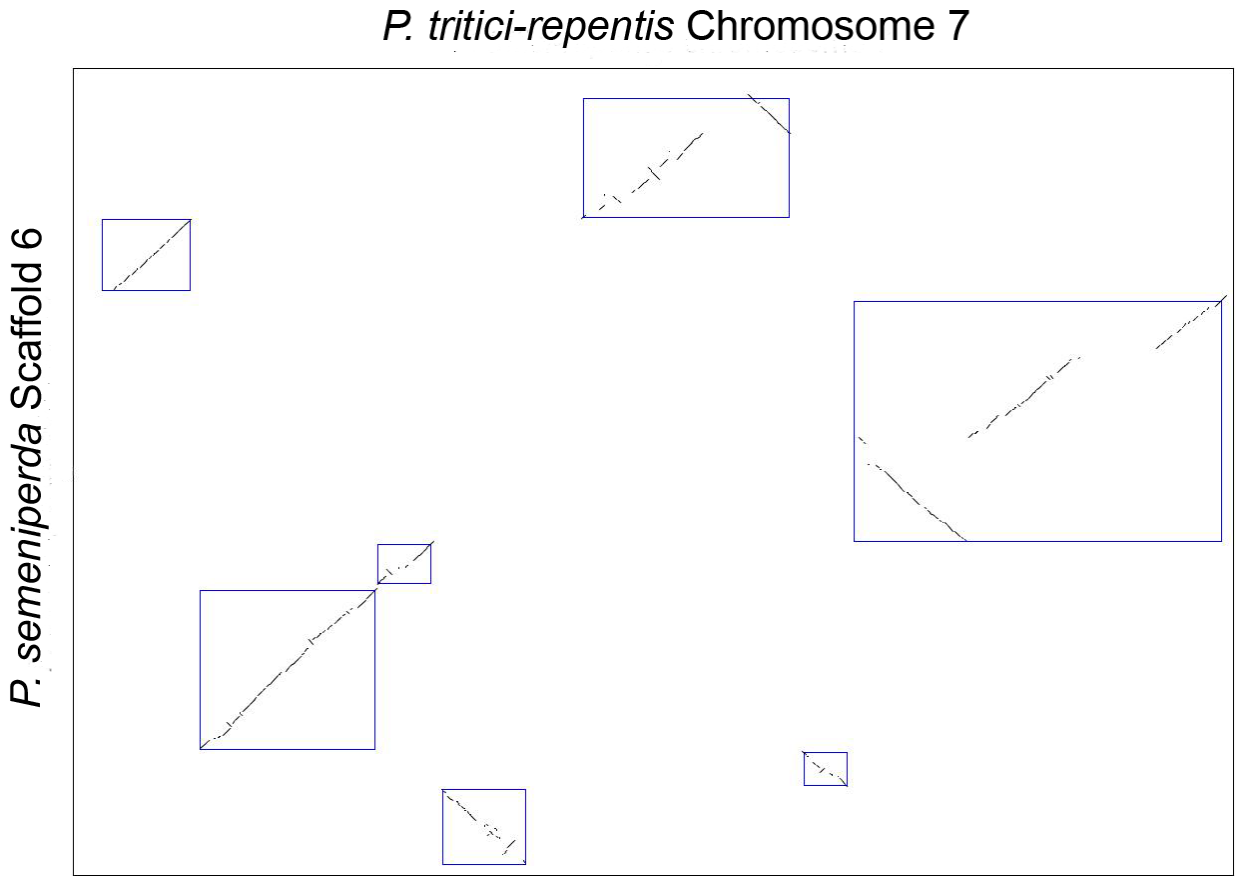
SyMap dot plot of *P. semeniperda* scaffold 6 and *P. tritici-repentis* chromosome 7. The dot plot alignment displays *P. tritici-repentis* chromosome 7 on the x-axis and *P. semeniperda* scaffold 6 on the y-axis. Boxes highlight regions of homology between the two genome regions.

**Figure 6:**
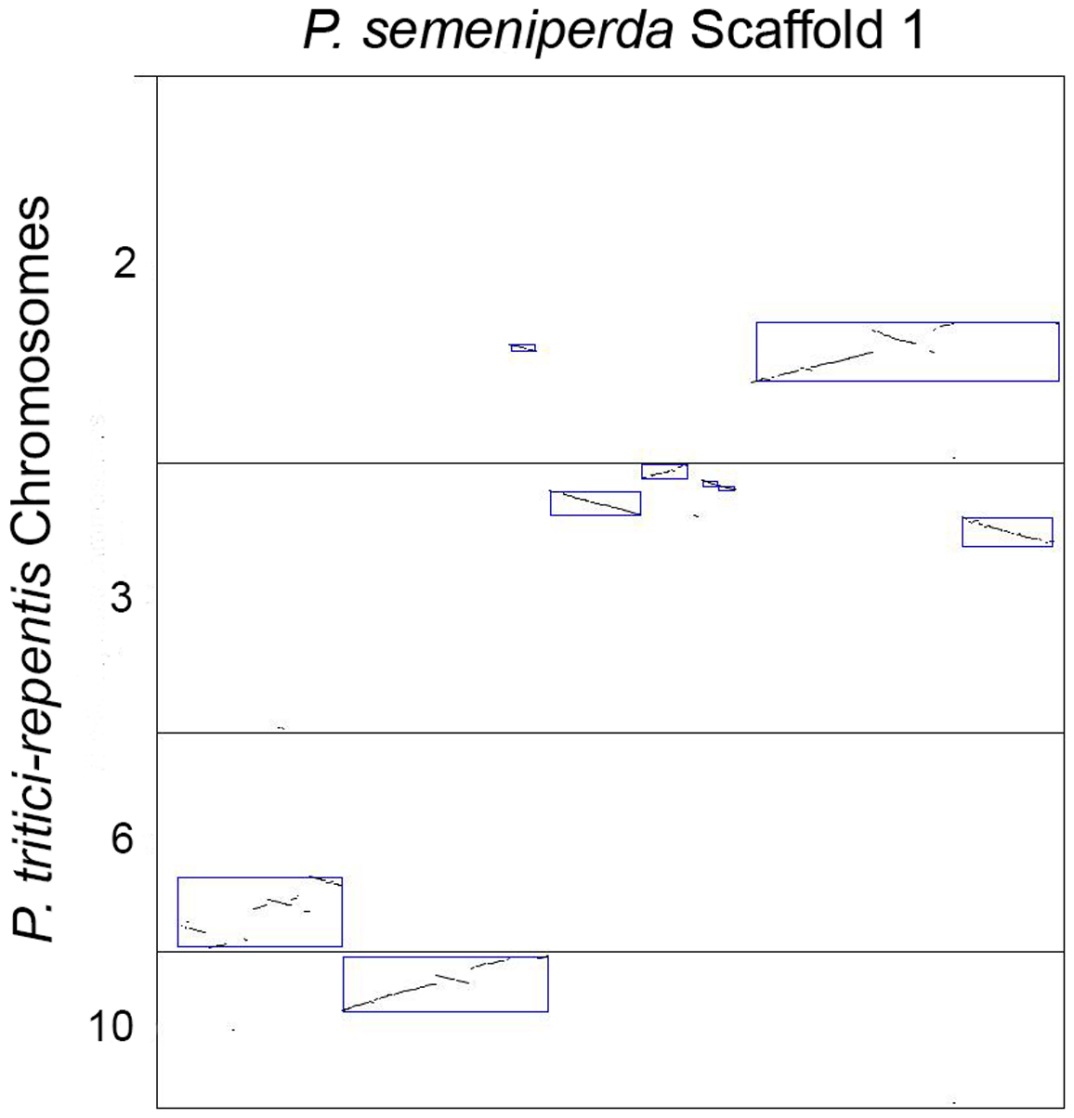
SyMap dot plot of alignment between *P. semeniperda* scaffold 1 and *P. tritici-repentis* 2, 3, 6 and 10. The *P. semeniperda* scaffold is displayed along the x-axis and the *P. tritici-repentis* chromosomes along the y-axis. Boxes highlight regions of homology between the two genomes.

A dot-plot of the genome alignment was created using Symap [27], revealing regions of synteny and colinearity between the two genome sequences (Figure 4). A total of 101 syntenic blocks with an identity range of 95% have been identified. Many of the observed rearrangements within the *P. semeniperda* scaffolds are localized within the corresponding *P. tritici-repentis* chromosome and include inversions, deletions, and transpositions (intrachromosomal rearrangements, Figure 5). These types of large-scale rearrangements are also observed when comparing the genomes of *Podospora anserina* and *Neurospora crassa*, most of which were intrachromosomal [51]–[53]. The distribution of intrachromosomal rearrangements is consistent across the *P. semeniperda* scaffolds with the exception of scaffold 1 which shows patterns of interchromosomal rearrangements, transposing to four different *P. tritici-repentis* chromosomes (Figure 6).

Large-scale genomic rearrangements such as those observed in *P. semeniperda* have been extensively studied in *S. cerevisiae*, many of which are attributed to recombination events between retrotransposons and other repetitive elements [42]. Further investigation of the *P. semeniperda* genome revealed transposable and repetitive elements flanking syntenic blocks suggesting that such elements may play a role in chromosomal rearrangements. Homology searches of these areas in *P. semeniperda* scaffolds reveal the presence of retroelements including *Ty1* and *Ty3*-type elements (copia and gypsy LTR elements), as well as *Gag*, *Env*, and *Pol* genes. *Ty* elements in *S. cerevisiae* have been shown to be sources of chromosomal crossovers which cause deletions, duplications, inversions, and translocations, though by what mechanisms and under what conditions this occurs under is unknown [42]. Additional molecular evidence is needed to make conclusions concerning the role retrotransposons have in the genomic architecture and evolution of *P. semeniperda*.

### Pathogenicity and infection-related genes

Because fungi use a variety of pathogenic strategies, it is not clear what mechanism is used by *P. semeniperda* to infect host seeds. To help identify putative infection mechanisms, the PHI-base fungal pathogenicity database were searched against the set of *P. semeniperda* gene models (tblastx, with an alignment threshold of ≤1×10^−20^). The PHI-base database contains 924 genes and their products from bacterial, fungi and oomycetes that have been demonstrated experimentally to be involved in pathogenesis [54]. The search identified 663 genes from *P. semeniperda* that matched 552 PHI-base entries (Table S1). Among the matches were putative genes that code for hydrolases, protease inhibitors, secondary metabolite biosynthesis enzymes, ABC transporters, and effector proteins, all factors related to virulence in necrotrophic plant pathogens. For instance, there are 19 genes in the *P. semeniperda* genome that encode proteins with homology to type I polyketide synthases. Fungal polyketides are important pharmacological compounds and are known virulence factors in several fungal species [55]. Other examples are 9 genes in the *P. semeniperda* genome that encode proteins with homology to cyclic peptide synthetases from *Alternaria alternata* and *Cochliobolus carbonum*. Cyclic peptides such as AM-toxin from *A. alternata* and HC-toxin from *C. carbonum* are important virulence factors whose synthesis is catalyzed, in part, by nonribosomal peptide synthetases [56]–[58].

### Secreted proteins

The expansion of secreted protein gene families has been observed in the genomes of the ascomycete phytopathogens *Stagnospora nodorum* and *Magnaporthe grisea* when compared with the saprophyte *Neurospora crassa* [59], [60], consistent with their role as plant pathogenic fungi. There are a relatively large number of putative genes encoding secreted proteins (996) in the *P. semeniperda* genome, as predicted by WolfP-SORT [61], ranging in length from 180–5,845 bp. A significant portion of the *P. semeniperda* secretome (81%) is homologous to *P. tritici-repentis* proteins. This level of homology is consistent with a similar analysis of the *P. teres* f. *teres* secretome [30], which shows that 85% of its predicted secreted proteins share homology with secreted proteins from *P. tritici-repentis*.

Nearly 55% (546 sequences) of the genes encoding secreted proteins were annotated with GO terms using Blast2GO [22], [23]. Although there are some drawbacks and limitations with the existing annotations databases due to their incompleteness [62], these GO terms provide a short synopsis of the types of secreted proteins that are found in *P. semeniperda*. Consistent with its role as a necrotrophic plant pathogen, many of the secreted proteins are putative enzymes that target various polysaccharides (Table 5). As observed in the previous assessment of pathogenic-related sequences described above, putative secreted proteins with hydrolase activity are homologous to proteins containing cellulose binding domains, carboxypeptidase, as well as cell wall glucanase and glycosyl hydrolase activity. Many of these sequences were also annotated with GO terms for oxidation reduction and oxidoreductase activity, suggesting that these gene products have key roles in the process of cellulose and lignin degradation [63].

**Table 5:** Common GO terms associated with secreted gene products.

GO identifier	Description	No. of genes
Molecular Function		
GO:0016787	Hydrolase activity	210
GO:0016491	Oxidoreductase activity	114
GO:0043167	Ion binding	97
GO:0016740	Transferase activity	56
GO:0000166	Nucleotide binding	54
GO:0048037	Cofactor binding	39
GO:0030246	Carbohydrate binding	28
GO:0005515	Protein binding	19
GO:0046906	Tetrapyrrole binding	17
GO:0001871	Pattern binding	17
GO:0016874	Ligase activity	11
GO:0016853	Isomerase activity	10
GO:0016829	Lyase activity	9
Biological process		
GO:0055114	Oxidation reduction	62
GO:0006508	Proteolysis	32
GO:0006629	Lipid metabolic process	27
GO:0043581	Mycelium development	26
GO:0050794	Regulation of cellular process	16
GO:0044248	Cellular catabolic process	13
GO:0006464	Protein modification process	13
GO:0006032	Chitin catabolic process	9
GO:0051591	Response to cAMP	8
GO:0045493	Xylan catabolic process	8

Molecular function GO terms are limited to level 3; GO term associated secretion gene products; adapted from Ellwood et al., 2010.

### Cytochalasin genes

Cytochalasins are a diverse group of fungal metabolites well-known for their ability to bind to actin filaments and block polymerization and elongation, thus inhibiting cytokinesis without affecting mitosis. Due to the ability of cytochalasins to block normal function of the cytoskeleton, many of them have been identified as antibiotic, antiviral, anti-inflammatory, or antitumoral agents [64]. Various cytochalasins forms have been identified in phytopathogenic fungi, including three previously unknown cytochalasins (Z1, Z2, and Z3) from *P. semeniperda* [65]. The exact role of cytochalasins in fungal virulence pathways is unknown; although, Beckstead et al. [16] suggest that *P. semeniperda* may use these compounds to inhibit germination of nondormant cheatgrass seeds and increase their vulnerability to attack from the fungus.

It is understood that the tricyclic ring system of cytochalasins is generated by a Diels-Alder-type reaction [66], [67]. Recently, the genes encoding the enzymes responsible for the early stages of cytochalasin biosynthesis in *Penicillium expansum* were identified by Schümann & Hertweck [64]. They identified 7 genes grouped together in what is now called the *chaetoglobosin* (*Che*) gene cluster. RNA silencing methods suggested that the *Che*A gene (encoding a PKS-NRPS hybrid protein) is essential to cytochalasin biosynthesis [64]. Using *Che* amino acid sequences from *P. expansum*, homologs of the genes in the *Che* cluster were found in the *P. semeniperda* genome sequence. Homologs were found for all seven genes including two PKS-NRPS protein genes. Like the *P. expansum* CheA protein, the putative *P. semeniperda* CheA proteins have PKS-NRPS hybrid domains as well as other protein features, including monooxygenase, transcription factor, and enoyl reductase domains. Putative *P. semeniperda Che* genes are not found in clusters as they are in *P. expansum* but are interspersed across multiple scaffolds.

### *Tox* Genes

Host-selective toxins (HSTs) have been identified in Pyrenophora species, specifically ToxA and ToxB HSTs in *Pyrenophora tritici-repentis*. These HSTs are proteinaceous effectors that are structurally unrelated and, though seem to evoke different host responses, confer the ability to cause disease in the host organism [68]. *P. tritici-repentis* races can be differentiated by their expression of one or any combination of tox genes which have all been shown to be pathogenic. A single copy of the *ToxA* gene in *P. tritici-repentis* is sufficient to induce necrosis on ToxA-sensitive wheat cultivars. Unlike *ToxA*, *ToxB*-containing isolates are more virulent with increasing *ToxB* gene copy numbers [69]–[71]. A correlation between *ToxB* transcript number and virulence/pathogenicity has been identified in *P. tritici-repentis*; the greater the *ToxB* transcript number, the more efficient it is able to cause disease in its host [70], [72].

A BLAST search using the *P. tritici repentis ToxA* gene sequence in a query of the *P. semeniperda* genome did not yield any hits; however a search using a *ToxB* query yielded a single copy in the *P. semeniperda* genome with 81% sequence similarity to the *P. tritici-repentis* sequence. Because *ToxB* and its homologs are primarily described as chlorosis-inducing toxins, its role in seed pathogenicity of P. semeniperda is currently unknown. Further study of *ToxB* copy number in other *P. semeniperda* isolates may produce a clearer understanding of its role in *P. semeniperda* virulence.

## Conclusions

The genome sequence, assembly and annotation of a single isolate of *P. semeniperda* are reported here. The assembly includes over 32 Mb with an estimated genome size of 40.1 Mb based on the metrics generated by the Newbler assembly. The size of the *P. semeniperda* genome is similar to the reported size of the *P. tritici-repentis* and *P. teres* genomes [10], [30], consistent with other related fungi. Genome comparisons between *P. semeniperda* and *P. tritici-repentis* allow visualization of large-scale rearrangements between these related species and provide clues to evolutionary mechanisms used by this fungus. The *P. semeniperda* genome contains a rich diversity of putative genes, common to other plant pathogens, notably hydrolases, ABC transporters, cytochrome P450 and secreted gene products attributable to other necrotrophs. In addition, the genome sequence can provide information for the development of molecular markers which may be implemented in population or evolutionary studies of this organism. This assembly also provides researchers with genomic and genetic resources to advance *P. semeniperda* research and the means to further our understanding of other phytopathogenic fungi.

The *P. semeniperda* genome is of immediate interest because of the genetic information it provides on putative genes that may play an important role in the infection process of the fungus on cheatgrass seeds. The genetic information is critical because it may inform efforts to create more powerful or effective fungal isolates to control the expansion of cheatgrass populations in the IMW. Future studies may include gene expression analyses that identify genes that are upregulated during the infection process. The genetic information will also make it possible to test the hypothesis that expression of *P. semeniperda* cytochalasin genes facilitates infection of nondormant seeds by inhibiting seed germination.

## Supporting Information

Table S1 *Pyrenophora semeniperda* gene models with homology to PHI-base protein entries.
(DOCX)
