# Supplementary material for: De novo Genome Assembly of the Fungal Plant Pathogen Pyrenophora semeniperda
Source: PLoS One. 2014 Jan 27;9(1):e87045. doi: 10.1371/journal.pone.0087045 (PMC3903604; doi:10.1371/journal.pone.0087045)
Supplement: Table S1 — Pyrenophora semeniperda gene models with homology to PHI-base protein entries. (DOCX) [file pone.0087045.s001.docx]

**Table S1.** *Pyrenophora semeniperda* gene models with homology to PHI-base protein entries.

| **Scaffold** | **Start** | **End** | **Putative Gene Description** | **Best PHI-Base Hit** |
| --- | --- | --- | --- | --- |
| 1 | 11272 | 9413 | MFS drug efflux transporter | 544 |
| 1 | 27581 | 24090 | cation-transporting ATPase | 440 |
| 1 | 63438 | 64799 | 3-ketoacyl-ketothiolase | 598 |
| 1 | 101803 | 102264 | cell division control protein | 281 |
| 1 | 322532 | 318377 | multidrug resistance protein | 1018 |
| 1 | 333565 | 332575 | adenyl cyclase-associated protein | 210 |
| 1 | 352018 | 353501 | β-glucansyltransferase | 33 |
| 1 | 374315 | 370152 | leptomycin B resistance protein | 1018 |
| 1 | 429119 | 431344 | carnithine O-acetyltransferase | 120, 594 |
| 1 | 461022 | 455740 | chitin synthase | 390 |
| 1 | 463308 | 468711 | chitin synthase | 389 |
| 1 | 518311 | 519555 | transcriptional regulator | 375 |
| 1 | 606340 | 609667 | glutamate decarboxylase | 816 |
| 1 | 609857 | 608177 | cell wall glucanase | 816 |
| 1 | 668146 | 669553 | ribonuclease T2 | 811 |
| 1 | 788852 | 789668 | C2H2 transcription factor | 439 |
| 1 | 858739 | 860500 | β-glucanosyltransferase | 522 |
| 1 | 942560 | 940547 | NADPH oxidase | 1024 |
| 1 | 992241 | 995168 | chitin synthase activator | 799 |
| 1 | 1106469 | 1109556 | white collar 1 blue-light regulator | 430 |
| 1 | 1220233 | 1221114 | appressorium specific protein | 256 |
| 1 | 1231824 | 1231070 | FAD-binding domain protein | 112 |
| 1 | 1283706 | 1284173 | formaldehyde dehydrogenase | 668 |
| 1 | 1289803 | 1288081 | alcohol dehydrogenase | 881 |
| 1 | 1308380 | 1311457 | 1-phosphatidylinositol-bisphosphate phosphodiesterase | 1026 |
| 1 | 1336214 | 1338222 | G_2_ mitotic-specific cyclin | 338 |
| 1 | 1391375 | 1393176 | phospholipase D | 373 |
| 1 | 1402716 | 1401022 | FAD bidning domain containing protein | 1046 |
| 1 | 1424974 | 1423051 | DNA polymerase δ catalytic subunit | 893 |
| 1 | 1444857 | 1443752 | alcohol dehydrogenase | 881 |
| 1 | 1654561 | 1653264 | G protein α subunit | 84 |
| 1 | 1671678 | 1670209 | tubulin γ chain | 823 |
| 1 | 1677966 | 1679229 | spermidine synthase | 384 |
| 1 | 1710940 | 1708607 | 4-coumarate ligase | 133 |
| 1 | 1763139 | 1761442 | ATP dependent RNA helicase | 423 |
| 1 | 1783166 | 1785748 | vacuolar sorting ATPase | 860 |
| 1 | 1899334 | 1898540 | naphthalenetriol reductase | 59 |
| 1 | 1921779 | 1928243 | polyketide synthase | 238 |
| 1 | 1935018 | 1936120 | glycoside hydrolase family 18protein | 144 |
| 1 | 2120920 | 2118925 | Zn(II)(2)Cys(6) cluster transcriptional activator | 169 |
| 1 | 2223749 | 2223940 | calcium clamodulin-dependent protein kinase | 1083 |
| 1 | 2229590 | 2228405 | mannitol 1-phosphate dehydrogenase | 413 |
| 1 | 2241416 | 2243021 | trans-aconitate 2-methyltransferase | 482 |
| 1 | 2329799 | 2328552 | voltage-gated chloride channel | 286 |
| 1 | 2339180 | 2340431 | carboxypeptidase | 901 |
| 1 | 2367781 | 2375754 | polyketide synthase | 55 |
| 1 | 2415982 | 2415482 | increased rDNA silencing protein | 488 |
| 1 | 2418251 | 2420866 | DNA ligase | 219 |
| 1 | 2436727 | 2434966 | mannose-6-phosphate isomerase | 220 |
| 1 | 2459179 | 2460444 | lipase | 541 |
| 1 | 2470799 | 2472113 | α-mannosyltransferase | 392 |
| 1 | 2572427 | 2571420 | tetrahydroxynaphthalene reductase | 59 |
| 1 | 2624743 | 2627793 | γ DNA polymerase | 505 |
| 1 | 2647283 | 2649116 | laccase-1 | 552 |
| 2 | 114869 | 116120 | serine-threonine protein phosphatase | 89 |
| 2 | 117383 | 119344 | carboxypeptidase | 901 |
| 2 | 212957 | 215492 | histone demethylation protein | 187 |
| 2 | 252288 | 244428 | polyketide synthase | 55 |
| 2 | 255649 | 257561 | isoamyl alcohol oxidase | 716 |
| 2 | 266521 | 262882 | histidine kinase response regulator | 599 |
| 2 | 366751 | 369333 | homoaconitase | 362 |
| 2 | 372089 | 372564 | secretion related GTPase | 339 |
| 2 | 391650 | 389950 | para-aminobenzoate synthase | 178 |
| 2 | 413576 | 412533 | cell surface protein | 257 |
| 2 | 525869 | 529576 | phospholipid-translocating P-type ATPase | 405 |
| 2 | 637429 | 638552 | endo-β-xylanase precursor | 569 |
| 2 | 671772 | 670339 | α-tubulin | 823 |
| 2 | 709965 | 707116 | GTP-binding protein | 512 |
| 2 | 723428 | 722391 | GTP-binding protein | 339 |
| 2 | 762855 | 759561 | plasma membrane H^+^-ATPase | 440 |
| 2 | 849048 | 845508 | mRNA binding protein | 882 |
| 2 | 854081 | 857301 | ethanolamine phosphate transferase | 259 |
| 2 | 1211765 | 1216113 | two-component histidine kinase | 550 |
| 2 | 1225856 | 1229036 | osmolarity two-component system protein | 140 |
| 2 | 1331659 | 1334677 | autoinducer sensor kinase/phosphatase | 837 |
| 2 | 1395379 | 1402066 | histidine kinase | 599 |
| 2 | 1447050 | 1444822 | glycoslytransferase | 243 |
| 2 | 1465599 | 1463686 | oligopeptide transporter | 1085 |
| 2 | 1512273 | 1513595 | endo-β-xylanase | 569 |
| 2 | 1550043 | 1553406 | α-glucosidase | 1071 |
| 2 | 1729972 | 1732665 | catalase | 1034 |
| 2 | 1755623 | 1754703 | enoyl-hydratase isomerase | 509 |
| 2 | 1815015 | 1807026 | NADPH oxidase | 1023 |
| 2 | 1834777 | 1830177 | copper-transporting ATPase | 244 |
| 2 | 1847608 | 1848558 | cutinase | 69 |
| 2 | 1862085 | 1864923 | urease | 194 |
| 2 | 1865996 | 1866505 | scytalone dehydratase | 58 |
| 2 | 1889082 | 1887168 | isocitrate lyase | 261 |
| 2 | 1904021 | 1902535 | peptidyl-prolyl cis-trans isomerase | 213 |
| 2 | 1919028 | 1922876 | ABC multidrug transporter | 1018 |
| 2 | 1938451 | 1938170 | protein kinase | 1072 |
| 2 | 1956310 | 1951593 | phospholipase | 373 |
| 2 | 1972774 | 1969637 | ethylene receptor | 599 |
| 2 | 1999080 | 2001285 | aspartyl protease | 891 |
| 2 | 2002149 | 2002617 | mitogen-activated protein kinase | 1032 |
| 2 | 2004234 | 2006160 | ATP-dependent RNA helicase | 423 |
| 2 | 2140080 | 2138307 | MFS multidrug transporter | 511 |
| 2 | 2147160 | 2145592 | mitotic spindle checkpoint component | 262 |
| 2 | 2157614 | 2164345 | MFS multidrug transporter | 510 |
| 2 | 2213636 | 2212806 | L-xyulose reductase | 1099, 1100 |
| 2 | 2216459 | 2214168 | MFS multidrug transporter | 737 |
| 2 | 2319075 | 2320588 | peroxisomal 3-ketoacyl-CoA thiolase | 598 |
| 2 | 2399472 | 2401163 | acetylcholinesterase precursor | 541 |
| 2 | 2408490 | 2410304 | α-glucosidase | 1071 |
| 2 | 2440105 | 2442006 | triacylglycerol lipase | 541 |
| 2 | 2466711 | 2465104 | serine-threonine protein kinase | 188 |
| 2 | 2474562 | 2477599 | chitin synthase | 1054 |
| 2 | 2498234 | 2499961 | 1,3-β-glucanosyltransferase | 33 |
| 2 | 2591500 | 2592694 | O-methyltransferase | 1051 |
| 2 | 2600786 | 2598030 | polyketide synthase | 40 |
| 3 | 30135 | 31955 | response regulator receiver | 189 |
| 3 | 45543 | 37920 | polyketide synthase | 55 |
| 3 | 46397 | 54267 | polyketide synthase | 713 |
| 3 | 147622 | 145754 | trehalose-phosphate synthase | 322, 1064 |
| 3 | 158423 | 161616 | MFS multidrug transporter | 544 |
| 3 | 167699 | 163974 | guanyl nucleotide exchange factor | 675 |
| 3 | 187563 | 185991 | methyltransferase | 482 |
| 3 | 196946 | 196946 | Ras small monomeric GTPase | 62 |
| 3 | 218353 | 218655 | superoxide dismutase | 330 |
| 3 | 327765 | 329738 | DNA photolyase | 1052 |
| 3 | 365571 | 372556 | polyketide synthase | 55 |
| 3 | 376273 | 373670 | UDP-transferase | 243 |
| 3 | 386462 | 384182 | extracellular dihydrogeodin oxidase laccase | 552 |
| 3 | 419536 | 417537 | pH-response transcription factor | 314 |
| 3 | 570202 | 567862 | xylanolytic transcriptional activator | 1022 |
| 3 | 588957 | 586260 | cell division protease | 226 |
| 3 | 615106 | 615804 | membrane transporter | 510 |
| 3 | 621685 | 624639 | serine-threonine protein kinase | 528 |
| 3 | 628368 | 629608 | peptidyl-prolyl cis-trans isomerase | 213 |
| 3 | 660355 | 661737 | class V chitinase | 144 |
| 3 | 715359 | 713752 | Key lime pathogenicity protein | 481 |
| 3 | 762296 | 761100 | chitin synthase activator | 799 |
| 3 | 764427 | 768221 | chitin synthase | 31 |
| 3 | 834366 | 830876 | histidine kinase | 562 |
| 3 | 843057 | 840210 | topoisomerase I | 80 |
| 3 | 849603 | 851081 | NADH-ubiquinone oxidoreductase | 445 |
| 3 | 909366 | 910862 | γ-butryobetaine hydroxylase | 792 |
| 3 | 922579 | 919529 | glycoside hydrolase | 191 |
| 3 | 995388 | 996656 | MFS multidrug transporter | 544 |
| 3 | 1076671 | 1075443 | copper resistance-associated protein | 244 |
| 3 | 1081911 | 1084224 | MFS peptide transporter | 1085 |
| 3 | 1151014 | 1146666 | peroxisomal biogenesis factor | 226 |
| 3 | 1154441 | 1156592 | α-tubulin | 823 |
| 3 | 1192631 | 1190573 | β-glucosidase | 816 |
| 3 | 1307162 | 1305085 | ATP-dependent RNA helicase | 423 |
| 3 | 1315937 | 1311006 | kinesin family protein | 465 |
| 3 | 1370618 | 1375185 | protein kinase | 1082 |
| 3 | 1381265 | 1378507 | serine-threonine protein kinase | 158 |
| 3 | 1396550 | 1395501 | 1-allo-threonine aldolase | 469 |
| 3 | 1456804 | 1458181 | 3-ketoacyl-thiolase | 598 |
| 3 | 1497600 | 1494950 | phospholipase C | 1026 |
| 3 | 1613032 | 1615527 | protease precursor | 306 |
| 3 | 1623359 | 1624709 | acetyl-acetyltransferase | 598 |
| 3 | 1711892 | 1713166 | multidrug resistance protein | 544 |
| 3 | 1747442 | 1749903 | methyltransferase | 482 |
| 3 | 1937019 | 1938821 | transcription factor | 1070 |
| 3 | 2003550 | 2006008 | mannosyltransferase | 399 |
| 3 | 2016608 | 2015190 | 26S protease regulatory subunit 7 | 226 |
| 3 | 2028426 | 2027227 | ribonuclease T2 | 811 |
| 4 | 62388 | 70570 | polyketide synthase | 55 |
| 4 | 128302 | 130980 | cytochrome c oxidase assembly | 503 |
| 4 | 210799 | 209041 | carboxypeptidase | 901 |
| 4 | 270915 | 268309 | phytochrome-like histidine kinase | 599 |
| 4 | 289529 | 291202 | serine threonine kinase | 1076 |
| 4 | 313694 | 311389 | ATP-dependent RNA helicase | 423 |
| 4 | 363029 | 364734 | FAD-binding domain containing protein | 1046 |
| 4 | 393418 | 390725 | ABC transporter | 1018 |
| 4 | 481622 | 479727 | MFS transporter | 511 |
| 4 | 568576 | 570530 | oligopeptide transporter | 1085 |
| 4 | 668349 | 669216 | cyanide hydratase | 143 |
| 4 | 673469 | 671702 | MFS transporter | 544 |
| 4 | 684270 | 682641 | cell cycle control protein | 280 |
| 4 | 759657 | 757597 | L-ascorbate oxidase | 397 |
| 4 | 790337 | 789864 | pectate lyase | 179 |
| 4 | 797366 | 795517 | laccase | 552 |
| 4 | 839262 | 841147 | multidrug resistance protein | 510 |
| 4 | 841510 | 847926 | MAP kinase | 296 |
| 4 | 848358 | 850422 | benomyl/methotrexate resistance protein | 510 |
| 4 | 859480 | 857488 | isoamyl alcohol oxidase | 716 |
| 4 | 903910 | 902369 | serine/threoning protein kinase | 360 |
| 4 | 963142 | 957994 | kinesin family protein | 464 |
| 4 | 1021415 | 1017741 | G_2_ mitotic-specific cyclin B | 338 |
| 4 | 1101756 | 1099242 | cutinase transcription factor | 1021 |
| 4 | 1140404 | 1138647 | β-tubulin | 820 |
| 4 | 1174151 | 1170320 | cell wall biogenesis protein phosphatase | 862 |
| 4 | 1202610 | 1199321 | protein kinase C | 376 |
| 4 | 1278544 | 1276996 | glucan β-glucosidase | 61 |
| 4 | 1283028 | 1281057 | MFS multidrug transporter | 511 |
| 4 | 1300042 | 1302825 | ATP-dependent RNA helicase | 423 |
| 4 | 1313510 | 1316037 | ATP-dependent RNA helicase | 423 |
| 4 | 1550872 | 1548964 | serine/threonine protein phosphatase | 378 |
| 4 | 1602053 | 1598405 | mitotic control protein | 862 |
| 4 | 1741225 | 1740410 | MFS multidrug transporter | 737 |
| 4 | 1748840 | 1752837 | pre-mRNA-processing ATP-dependent RNA helicase | 423 |
| 4 | 2012226 | 2008520 | ABC transporter | 202 |
| 5 | 79864 | 80882 | N-acetylglucsamine-6-phosphate deacetylase | 207 |
| 5 | 122043 | 123901 | triacylclycerol lipase | 541 |
| 5 | 172909 | 175275 | glycoside hydrolase | 191 |
| 5 | 279169 | 281090 | amidophosphoribosyltransferase | 502 |
| 5 | 359800 | 361864 | ATP-depedent RNA helicase | 423 |
| 5 | 379008 | 378325 | fructose facilitator | 511 |
| 5 | 440856 | 439320 | FAD-binding domain containing protein | 1046 |
| 5 | 456275 | 455403 | glucosamine 6-phosphate N-acetyl transferase | 174 |
| 5 | 474189 | 475020 | tetraspanin | 429 |
| 5 | 480977 | 485774 | phospholipid-translocating P-type ATPase | 405 |
| 5 | 511256 | 509010 | glycoside hydrolase | 748 |
| 5 | 513382 | 511461 | citrate synthase | 447 |
| 5 | 514042 | 514946 | isocitrate lyase | 477 |
| 5 | 536460 | 532054 | mitochondrial ATPase | 860 |
| 5 | 563273 | 562062 | argininosuccinate lyase | 200 |
| 5 | 624371 | 627111 | transcriptional corepressor | 320 |
| 5 | 642008 | 639004 | nitrogen regulatory protein | 287 |
| 5 | 650477 | 649598 | sugar transporter | 538 |
| 5 | 720845 | 719170 | malate synthase | 365 |
| 5 | 734135 | 734495 | fatty acid oxygenase | 494 |
| 5 | 772642 | 769391 | DNA repair protein | 890 |
| 5 | 835905 | 837869 | choline dehydrogenase | 922 |
| 5 | 896658 | 894778 | siderophore iron transporter | 513 |
| 5 | 941355 | 935497 | noribosomal peptide synthase | 1008 |
| 5 | 947296 | 942524 | leptomycin B resistance protein | 1018 |
| 5 | 948960 | 947827 | L-ornithine monooxygenase | 377, 486 |
| 5 | 1018099 | 1017253 | transcription factor | 412 |
| 5 | 1069269 | 1068268 | kinesin heavy chain | 465 |
| 5 | 1246063 | 1248817 | serine/threonine protein kinase | 36 |
| 5 | 1260207 | 1259247 | manganese superoxide dismutase | 401 |
| 5 | 1290919 | 1289633 | tartrate dehydrogenase | 504 |
| 5 | 1297981 | 1294727 | cation transport ATPase | 244 |
| 5 | 1332827 | 1331772 | nitrilase | 143 |
| 5 | 1352501 | 1350789 | FAD-binding domain containing protein | 1046 |
| 5 | 1409067 | 1406782 | cell division protease | 226 |
| 5 | 1435198 | 1436408 | flavohemoprotein | 667 |
| 5 | 1454863 | 1456338 | guanine nucleotide-binding protein α-3 subunit | 75 |
| 5 | 1457364 | 1459352 | short chain dehydrogenase reductase | 1099, 1100 |
| 5 | 1471955 | 1466765 | ABC transporter | 132 |
| 5 | 1478618 | 1483284 | rho guanylnucleotide exchange factor | 283 |
| 5 | 1503005 | 1502460 | aldo-keto reductase | 419 |
| 5 | 1522446 | 1520822 | lysophospholipase | 105 |
| 5 | 1641970 | 1644290 | chitin synthase | 565 |
| 5 | 1706230 | 1709081 | vacuolar ATP synthase | 235 |
| 5 | 1737335 | 1738066 | FAD-binding domain containing protein | 1046 |
| 5 | 1747698 | 1752607 | ABC multidrug transporter | 1030 |
| 5 | 1753443 | 1756270 | phosphoenol pyruvate carboxykinase | 424 |
| 5 | 1783557 | 1782996 | bZIP transcription factor | 344 |
| 6 | 63584 | 66485 | homoserine O-acetyltransferase | 355 |
| 6 | 95566 | 98323 | peroxiredoxin | 386 |
| 6 | 112625 | 110901 | β-D-glucoside glucohydrolase | 748 |
| 6 | 116947 | 118001 | cell division control protein | 449 |
| 6 | 178158 | 179848 | alcohol dehydrogenase | 881 |
| 6 | 180179 | 183044 | voltage-gated chloride channel | 286 |
| 6 | 192201 | 193928 | isoamyl alcohol oxidase | 716 |
| 6 | 253282 | 251914 | peptidyl-prolyl cis-trans isomerase | 436 |
| 6 | 262885 | 259511 | GTP-binding protein | 124 |
| 6 | 272116 | 271180 | integral membrane protein | 404 |
| 6 | 277419 | 281274 | multidrug resistance protein | 202 |
| 6 | 360617 | 359953 | endo-β-xylanase | 572 |
| 6 | 453805 | 453054 | heat-stable 19-kDa antigen precursor | 695 |
| 6 | 479787 | 477759 | DSB repair complex subunit | 1038, 1039 |
| 6 | 540158 | 539314 | homoserine O-acetyltransferase | 355 |
| 6 | 567102 | 568505 | para-nitrobenzyl esterase | 541 |
| 6 | 659470 | 661047 | para-nitrobenzyl esterase | 541 |
| 6 | 665614 | 668460 | phospholipid transporting ATPase | 405 |
| 6 | 690567 | 692414 | MFS membrane transporter | 737 |
| 6 | 729355 | 730838 | oligopeptide transporter | 1085 |
| 6 | 735523 | 732976 | voltage-gated chloride channel | 286 |
| 6 | 769035 | 769784 | sorbose reductase | 1099, 1100 |
| 6 | 775585 | 773006 | kinesin motor domain-containing protein | 465 |
| 6 | 792657 | 791355 | nuclear division protein | 781 |
| 6 | 806217 | 805855 | squalene monooxygenase | 825 |
| 6 | 900443 | 897846 | phosphoinositide 3-kinase | 195 |
| 6 | 921932 | 923349 | MFS monocarboxylate transporter | 812 |
| 6 | 944303 | 945352 | FAD-binding domain protein | 112 |
| 6 | 952360 | 953222 | α-mannosyltransferase | 104 |
| 6 | 999429 | 1000544 | endo-β-xylanase precursor | 568 |
| 6 | 1020275 | 1023795 | eukaryotic initiation factor | 423 |
| 6 | 1076493 | 1074772 | ceramide glucosyltransferase | 693 |
| 6 | 1141698 | 1140292 | β-glucanosyltransferase | 434 |
| 6 | 1165238 | 1162831 | sugar transporter | 538 |
| 6 | 1242215 | 1239790 | serine/threonine protein kinase | 518 |
| 6 | 1319587 | 1318535 | α-mannsoyltransferase | 392 |
| 6 | 1400553 | 1404526 | ABC transporter | 391 |
| 6 | 1459473 | 1461191 | MFS transporter | 544 |
| 6 | 1512236 | 1510515 | FAD-binding domain protein | 1046 |
| 6 | 1532657 | 1535814 | α-glucosidase | 1071 |
| 6 | 1544504 | 1542840 | sodium transport ATPase | 440 |
| 6 | 1570613 | 1572007 | cell division control protein | 281 |
| 6 | 1594547 | 1592765 | MFS drug transporter | 544 |
| 6 | 1660755 | 1658442 | serine threonine protein phosphatase 2b catalytic subunit | 89 |
| 6 | 1695147 | 1689981 | histone demethylase | 187 |
| 7 | 84569 | 83160 | cAMP-dependent protein kinase | 231 |
| 7 | 253644 | 250688 | two-component histidine protein kinase sensor | 599 |
| 7 | 310797 | 312446 | carboxypeptidase | 901 |
| 7 | 344907 | 345866 | acetyl-CoA-acetyltransferase | 598 |
| 7 | 408438 | 406038 | neutral trehalase | 651 |
| 7 | 449707 | 444316 | polyketide synthase | 101 |
| 7 | 461892 | 463221 | 26S protease regulatory subunit S10b | 226 |
| 7 | 470221 | 466939 | serine/threonine protein kinase | 109 |
| 7 | 508182 | 503861 | ABC transporter | 202 |
| 7 | 529887 | 530969 | cellulose-β-cellobiosidase | 566 |
| 7 | 639482 | 636534 | small nucleolar ribonucleoprotein complex subunit | 211 |
| 7 | 643931 | 646666 | kinesin | 464 |
| 7 | 675256 | 670561 | ABC transporter | 1030 |
| 7 | 689623 | 687817 | MFS transporter | 544 |
| 7 | 694288 | 692611 | MFS multidrug transporter | 511 |
| 7 | 731223 | 733823 | centromere kinetochore protein | 873 |
| 7 | 820750 | 819649 | branched-chain amino acid aminotransferase | 157 |
| 7 | 838621 | 837681 | ornithine decarboxylase | 177 |
| 7 | 904571 | 904834 | peptidylprolyl cis-trans isomerase | 548 |
| 7 | 958840 | 959927 | splicing factor | 801 |
| 7 | 965198 | 962587 | thermostable β-glucosidase B | 191 |
| 7 | 1009289 | 1009939 | mitochondrial succinate dehydrogenase iron-sulfur subunit | 822 |
| 7 | 1049453 | 1047386 | ATP-dependent RNA helicase | 423 |
| 7 | 1066759 | 1063404 | fatty acid oxygenase | 494 |
| 7 | 1129059 | 1131122 | pH response protein | 186 |
| 7 | 1350552 | 1345676 | ABC multidrug transporter | 267 |
| 7 | 1430872 | 1429398 | 14α-sterol demethylase | 838 |
| 7 | 1433989 | 1431643 | serine/threonine protein kinase | 1083 |
| 7 | 1451020 | 1452507 | prenyl protease | 807 |
| 7 | 1572399 | 1575780 | calcium-translocating P-type ATPase | 440 |
| 7 | 1583337 | 1584941 | pathogenicity protein | 597 |
| 7 | 1611848 | 1610291 | nucleoside dephosphatase | 781 |
| 7 | 1624922 | 1633308 | MFS quinate transporter | 876 |
| 8 | 46550 | 44171 | catalase | 1034 |
| 8 | 58627 | 60303 | C2H2transcription factor | 795 |
| 8 | 123941 | 119202 | UDP-glucose:glycoprotein glucosyltransferase | 361 |
| 8 | 137019 | 144925 | polyketide synthase | 55 |
| 8 | 201303 | 197017 | serine/threonine protein kinase | 188 |
| 8 | 261011 | 262001 | rho GTPase | 270 |
| 8 | 311601 | 312743 | MFS aflatoxin efflux pump | 544 |
| 8 | 436701 | 428392 | lovastatin nonaketide synthase | 325 |
| 8 | 439410 | 437681 | 6-hydroxy-D-nicotine oxidase | 716 |
| 8 | 447531 | 448610 | isoamyl alcohol oxidase | 716 |
| 8 | 453799 | 452663 | MFS gliotoxin efflux transporter | 544 |
| 8 | 490568 | 488160 | 5-methyltetrahydropteroyltriglutamate-homocysteine methyltransferase | 442 |
| 8 | 494427 | 492763 | FAD binding domain containing protein | 1046 |
| 8 | 510300 | 508911 | plastidic glucose transporter | 538 |
| 8 | 662956 | 665176 | developmental regulator | 345 |
| 8 | 670374 | 667321 | serine/threonine protein kinase | 1058 |
| 8 | 768134 | 766957 | cell division control protein | 281 |
| 8 | 780057 | 781319 | heavy metal tolerance protein | 1018 |
| 8 | 782871 | 787472 | nonribosomal peptide synthase-like protein | 160 |
| 8 | 953277 | 954491 | CFEM domain-containing protein | 404 |
| 8 | 1093000 | 1092126 | endochitinase precursor | 144 |
| 8 | 1387115 | 1389602 | avenacinase-like protein | 24 |
| 8 | 1445323 | 1443420 | MFS alfatoxin efflux pump | 544 |
| 8 | 1452344 | 1453023 | scytalone dehydratase | 58 |
| 8 | 1459054 | 1453516 | polyketide synthase | 40 |
| 8 | 1459579 | 1463250 | 17-β-hydroxysteroid dehyrdogenase | 59 |
| 9 | 41181 | 42838 | serine/threonine protein kinase | 158 |
| 9 | 72096 | 76283 | serine/threonine protein kinase | 301 |
| 9 | 79509 | 80128 | NADP-dependent alcohol dehydrogenase C | 881 |
| 9 | 159134 | 157317 | peptidase aspartic | 891 |
| 9 | 171743 | 173315 | hypothetical protein | 885 |
| 9 | 188699 | 187746 | alcohol dehydrogenase | 881 |
| 9 | 193266 | 191470 | MAPKK kinase | 1073 |
| 9 | 322806 | 324842 | glucose-methanol-choline oxidoreductase | 922 |
| 9 | 419931 | 417676 | isoamyl oxidase | 716 |
| 9 | 434149 | 433190 | protein kinase regulator | 1074 |
| 9 | 439576 | 437443 | cell division control protein cdc48 | 226 |
| 9 | 510728 | 508806 | MFS sugar transporter | 538 |
| 9 | 537383 | 540067 | 1-phosphatidylinositol-4,5-bisphosphate phosphodiesterase gamma 2 | 1026 |
| 9 | 593363 | 589530 | cell division control protein cdc25 | 319 |
| 9 | 683344 | 684558 | G protein α subunit | 353 |
| 9 | 782607 | 785920 | histidine kinase | 551 |
| 9 | 1043704 | 1042466 | flavoprotein monooxygenase | 1048 |
| 9 | 1047132 | 1047881 | peptidylprolyl cis-trans isomerase | 249 |
| 9 | 1182154 | 1180495 | kinesin | 465 |
| 9 | 1237997 | 1236834 | chitin synthase export chaperone | 337 |
| 9 | 1272360 | 1256055 | non-ribosomal peptide synthase | 416 |
| 9 | 1273515 | 1276630 | ABC bile acid transporter | 267 |
| 9 | 1318827 | 1320218 | serine palmitoyltransferase | 595 |
| 9 | 1338536 | 1339674 | G protein complex β subunit | 334 |
| 9 | 1351268 | 1355800 | phospholipid transporting ATPase | 405 |
| 9 | 1380485 | 1383394 | α-glucosidase | 526, 1071 |
| 10 | 83044 | 80285 | SPX domain-containing protein | 790 |
| 10 | 222147 | 222974 | methyltransferase | 482 |
| 10 | 225344 | 224455 | superoxide mitochondrial precursor | 410 |
| 10 | 307420 | 302906 | ABC multidrug transporter | 267 |
| 10 | 322940 | 316827 | fatty acid synthase | 97 |
| 10 | 328923 | 324367 | fatty-acid synthase α subunit reductase | 96 |
| 10 | 335754 | 348252 | nonribosomal peptide synthase | 12 |
| 10 | 376849 | 375110 | aryl-alcohol dehydrongenase | 922 |
| 10 | 478075 | 475672 | glycosyltransferase | 243 |
| 10 | 483706 | 481261 | plasma membrane calcium-transporting ATPase | 440 |
| 10 | 521849 | 520865 | cell cycle regulatory protein | 346 |
| 10 | 560284 | 561431 | peroxisome biosynthesis protein | 797 |
| 10 | 578868 | 581141 | iron-sulfur clusters transporter | 1018 |
| 10 | 593248 | 594302 | glycoside hydrolase | 547 |
| 10 | 599179 | 600812 | benzoate 4-monooxygenase cytochrome p450 | 438 |
| 10 | 610719 | 608003 | DNA mismatch repair protein | 872 |
| 10 | 644171 | 643146 | tyrosine protein phosphatase | 491 |
| 10 | 762955 | 766156 | Ras guanine-nucleotide exchange protein | 319 |
| 10 | 925678 | 922685 | trehalose-phosphate synthase subunit | 242 |
| 10 | 953804 | 951395 | aureobasidin resistance protein | 218 |
| 10 | 963453 | 965165 | 4-coumarate-CoA ligase | 508 |
| 10 | 1033520 | 1031782 | peroxisomal catalase | 106 |
| 10 | 1083401 | 1084856 | MFS gliotoxin efflux transporter | 544 |
| 10 | 1138216 | 1140040 | septin | 282 |
| 10 | 1231734 | 1233090 | DUF1237 domain-containing protein | 785 |
| 10 | 1332124 | 1325721 | MIT domain-containing protein | 815 |
| 10 | 1341393 | 1340250 | alcohol dehydrogenase | 881 |
| 10 | 1359810 | 1354866 | ABC transporter | 267 |
| 10 | 1364043 | 1386354 | nonribosomal peptide synthase | 12 |
| 11 | 194864 | 192881 | choline dehydrogenase | 922 |
| 11 | 201398 | 202694 | 2-ketoacyl-thiolase | 598 |
| 11 | 253041 | 251824 | trimethyllysine dioxgenase | 774, 792 |
| 11 | 254646 | 256158 | 26S protease regulatory subunit 6A | 226 |
| 11 | 332662 | 330510 | acetolactate synthase | 358 |
| 11 | 421763 | 422340 | rho GTPase | 270 |
| 11 | 525304 | 526908 | ATP-dependent RNA helicase | 423 |
| 11 | 701285 | 702285 | SAM-dependent methyltransferase | 482 |
| 11 | 738687 | 740524 | carboxylesterase | 541 |
| 11 | 776831 | 773038 | cell division control protein | 791 |
| 11 | 970144 | 972004 | cytochrome p450 monooxygenase | 438 |
| 11 | 1109666 | 1110965 | cell division cycle protein 48 | 226 |
| 11 | 1139644 | 1123531 | non-ribosomal peptide synthase | 160 |
| 11 | 1141159 | 1145414 | leptomycin B resistance protein | 1018 |
| 11 | 1146885 | 1149060 | L-ornithine N5-oxygenase | 486 |
| 11 | 1150433 | 1151633 | protein kinase | 149 |
| 11 | 1193720 | 1187408 | fatty acid synthase β-subunit dehydratase | 97 |
| 11 | 1195092 | 1200819 | fatty acid synthase α subunit | 96 |
| 11 | 1203292 | 1204605 | mannitol 1-phosphate dehydrogenase | 413 |
| 11 | 1223352 | 1225097 | isoamyl alcohol | 716 |
| 11 | 1226734 | 1225706 | FAD-binding domain protein | 716 |
| 11 | 1236788 | 1249983 | polyketide synthase/peptide synthetase | 325 |
| 11 | 1277736 | 1276864 | MFS transporter | 812 |
| 11 | 1305454 | 1302446 | RNAPII degradation factor | 805 |
| 11 | 1340403 | 1342661 | NADPH oxidase regulator | 1025 |
| 11 | 1363154 | 1365487 | serine/threonine protein kinase | 376 |
| 12 | 4760 | 4251 | HC-toxin synthetase | 160 |
| 12 | 8635 | 6141 | HC-toxin synthetase | 160 |
| 12 | 9493 | 10922 | HC-toxin synthetase | 160 |
| 12 | 15564 | 17456 | nonribosomal peptide synthase | 416 |
| 12 | 41662 | 40002 | peptide transporter | 1085 |
| 12 | 66310 | 72192 | adenylate cyclase | 332 |
| 12 | 77676 | 76380 | zeaxanthin epoxidase | 413 |
| 12 | 110110 | 117904 | C6 zinc finger domain containing protein | 889 |
| 12 | 236375 | 238689 | calcium-transporting ATPase | 440 |
| 12 | 298756 | 296404 | alcohol oxidase | 199 |
| 12 | 301691 | 300704 | alternative oxidase | 275 |
| 12 | 367568 | 369461 | nonribosomal peptide synthase | 160 |
| 12 | 420152 | 418187 | sulfate adenylyltransferase | 265 |
| 12 | 498592 | 500364 | glycylpeptide N-tetradecanoyltransferase | 19 |
| 12 | 551199 | 550312 | benomyl methotrexate resistance protein | 26 |
| 12 | 600565 | 595570 | ATPase family AAA domain-containing protein | 226 |
| 12 | 653564 | 658168 | DNA polymerase ζ catalytic subunit | 893 |
| 12 | 749802 | 748518 | MFS multidrug transporter | 544 |
| 12 | 803855 | 804485 | GTP-binding protein rho3 | 1061 |
| 12 | 829101 | 830733 | serine/threonine protein phosphatase | 378 |
| 12 | 958292 | 956661 | MFS monocarboxylate transporter | 812 |
| 12 | 987421 | 988577 | monocarboxylate permease | 812 |
| 12 | 998568 | 1000277 | phosphatase | 923 |
| 12 | 1003649 | 1001590 | glyoxal oxidase | 352 |
| 12 | 1068270 | 1071617 | β-glucosidase | 748 |
| 12 | 1094401 | 1093228 | lipase | 432 |
| 12 | 1179714 | 1178103 | class V chitinase | 144 |
| 12 | 1214079 | 1217850 | MAP kinase | 246, 266 |
| 12 | 1283337 | 1278767 | ABC multidrug resistance protein | 1018 |
| 13 | 7482 | 9224 | cytochrome P450 | 838, 841 |
| 13 | 29118 | 30714 | serine/threonine protein phosphatase | 378 |
| 13 | 132876 | 133680 | ras protein | 182 |
| 13 | 207825 | 208683 | MFS transporter | 511 |
| 13 | 370915 | 372797 | MFS multidrug transporter | 544 |
| 13 | 390508 | 392279 | 1,3-β-glucanosyltransferase | 522 |
| 13 | 474607 | 470656 | lipid A export ATP-binding/permease protein | 1018 |
| 13 | 491694 | 489780 | FAD-binding domain containing protein | 716 |
| 13 | 494320 | 492444 | alcohol oxidase | 922 |
| 13 | 550157 | 553003 | hypothetical protein | 783 |
| 13 | 567106 | 570838 | P-type calcium ATPase | 440 |
| 13 | 584740 | 585359 | GTP binding protein rho4 | 270 |
| 13 | 599919 | 601345 | exoglucanase | 566 |
| 13 | 651973 | 653615 | glycolipid 2-α-mannosyltransferase | 392 |
| 13 | 742350 | 741565 | endo-β-xylanase | 571 |
| 13 | 769376 | 768140 | alcohol dehydrogenase | 881 |
| 13 | 772248 | 771045 | CTLH-domain containing protein | 806 |
| 13 | 774892 | 774221 | serine/threonine protein phosphatase | 378 |
| 13 | 847870 | 846598 | DUF1237 domain containing protein | 785 |
| 13 | 863610 | 864792 | aldo keto reductase | 419 |
| 13 | 870302 | 873297 | ATPase 60S ribosome export protein | 593 |
| 13 | 899475 | 897800 | FAD-binding domain protein | 1046 |
| 13 | 935816 | 936766 | glucose-methanol-choline oxidoreductase | 922 |
| 13 | 952388 | 950865 | FAD-binding domain containing protein | 716 |
| 13 | 973623 | 971959 | serine/threonine protein phosphatase | 378 |
| 13 | 1069425 | 1066773 | xylanolytic transcriptional activator | 1022 |
| 13 | 1122567 | 1124100 | 1,3-β-glucanosyltransferase | 434 |
| 13 | 1155054 | 1152208 | peroxisome biosynthesis protein | 226 |
| 13 | 1183902 | 1185623 | endo-β-xylanase | 569 |
| 14 | 58050 | 58571 | orotate phosphoribosyltransferase | 159 |
| 14 | 168839 | 172026 | ABC transporter | 267 |
| 14 | 197499 | 196490 | FAD-binding domain containing protein | 1046 |
| 14 | 208296 | 212092 | SH3 domain-containing protein | 789 |
| 14 | 219436 | 220327 | citrinin biosynthesis transporter | 737 |
| 14 | 274129 | 273239 | flavoprotein monooxygenase | 1048 |
| 14 | 323740 | 320609 | histidine kinase | 253 |
| 14 | 329059 | 327932 | hypothetical protein | 888 |
| 14 | 336148 | 332650 | chitin synthase | 336 |
| 14 | 400309 | 398661 | ATPase | 860 |
| 14 | 489403 | 490330 | acetylcholinesterase | 541 |
| 14 | 577715 | 576426 | polygalacturonase | 114 |
| 14 | 588339 | 593204 | ABC multidrug transporter | 310 |
| 14 | 595424 | 597982 | β-glucosidase | 24 |
| 14 | 603443 | 604084 | carbohydrate esterase | 278, 1028 |
| 14 | 625415 | 623547 | alcohol oxidase | 199 |
| 14 | 679055 | 677889 | endopolygalacturonase | 103, 1027 |
| 14 | 696306 | 697990 | cystathionine β-lyase | 443 |
| 14 | 793494 | 795350 | FAD-binding domain containing protein | 716 |
| 14 | 800932 | 799481 | cytochrome p450 | 438 |
| 14 | 805489 | 802704 | 3-isopropylmalate dehydratase | 362 |
| 14 | 841788 | 841025 | 3-hydroxybenzoate 6-hydroxylase | 112 |
| 14 | 846954 | 847487 | peptidylprolyl isomerase | 249 |
| 14 | 924855 | 927060 | cutinase transcription factor | 1021 |
| 14 | 964746 | 970871 | polyketide synthase | 55 |
| 14 | 1022012 | 1023111 | vacuolar ATP synthase | 435 |
| 15 | 121675 | 119760 | peptide transporter | 1085 |
| 15 | 135737 | 136491 | peptidyl-prolyl cis-trans isomerase | 277 |
| 15 | 273382 | 274517 | pectate lyase | 180 |
| 15 | 309836 | 313435 | 3-hydroxy-3-methylglutaryl-coenzyme A reductase | 1006 |
| 15 | 316530 | 314722 | calcium calmoldulin-dependent protein kinase | 1082 |
| 15 | 339737 | 338179 | sugar transporter | 538 |
| 15 | 374087 | 375459 | pyrazinamidase nicotinamidase | 1006 |
| 15 | 409035 | 407750 | mannitol 1-phosphate dehydrogenase | 413 |
| 15 | 481264 | 482484 | ER lumen protein retaining receptor | 782, 796 |
| 15 | 531365 | 534343 | vacuolar ABC heavy metal transporter | 1018 |
| 15 | 537422 | 539322 | histone deacetylase | 216 |
| 15 | 569292 | 570741 | integral membrane protein | 803 |
| 15 | 605005 | 604523 | necrosis and ethylene-producing protein | 741 |
| 15 | 646380 | 648167 | D-arabino-lactone oxidase | 197 |
| 15 | 650192 | 649389 | endo-β-xylanase | 572 |
| 15 | 766320 | 765136 | calcineurin subunit B | 474 |
| 15 | 790926 | 789014 | MAP kinase kinase | 402 |
| 15 | 802647 | 800519 | protein kinase | 113 |
| 15 | 832442 | 834178 | polyamine transporter | 26 |
| 16 | 20874 | 23413 | 3-isopropylmalate dehydrogenase | 504 |
| 16 | 30261 | 28785 | carboxypeptidase Y | 901 |
| 16 | 95912 | 94507 | MFS multidrug transporter | 511 |
| 16 | 104641 | 102644 | cAMP-specific phophodiesterase | 857 |
| 16 | 162280 | 160279 | ATP-dependent RNA helicase | 423 |
| 16 | 172960 | 171667 | plasma membrane iron permease | 162, 485 |
| 16 | 173985 | 175892 | iron transport multicopper oxidase | 397 |
| 16 | 181084 | 181929 | orotidine-5-phosphate decarboxylase | 506 |
| 16 | 403559 | 403951 | imidazoleglycerol-phosphate dehydratase | 121 |
| 16 | 429434 | 430521 | transcriptional repressor | 211 |
| 16 | 452512 | 450643 | 5-aminolevulinate synthase | 595 |
| 16 | 510210 | 509332 | MFS monocarboxylate transporter | 812 |
| 16 | 655353 | 654376 | β-glucanase | 323 |
| 16 | 721928 | 723683 | isoamyl alcohol oxidase | 716 |
| 16 | 733425 | 731572 | benomyl methotrexate resistance protein | 26 |
| 16 | 823061 | 824107 | MFS transporter | 510 |
| 16 | 828869 | 829150 | polyketide synthase | 325 |
| 17 | 5050 | 3350 | phosphoribosylaminoimidazole carboxylase | 196 |
| 17 | 62673 | 61164 | C2H2 transcription factor | 331 |
| 17 | 104294 | 102911 | S-adenosylmethionine synthetase | 877 |
| 17 | 120570 | 115872 | HET-S domain protein | 800 |
| 17 | 187567 | 189382 | MAP kinase kinase | 1072 |
| 17 | 321414 | 319644 | carboxypeptidase | 903 |
| 17 | 328738 | 327326 | Class V chitinase | 144 |
| 17 | 336003 | 336464 | endopolygalacturonase | 3 |
| 17 | 395357 | 397739 | laccase-1 | 552 |
| 17 | 465769 | 467476 | ATP-dependent RNA helicase | 423 |
| 17 | 500644 | 503658 | β-glucosidase | 748 |
| 17 | 615302 | 613343 | sodium transport ATPase | 440 |
| 18 | 276480 | 275332 | protein kinase | 519 |
| 18 | 307635 | 307305 | small monomeric GTPase | 317 |
| 18 | 366805 | 368734 | striatin | 731 |
| 18 | 450340 | 445101 | ABC metal ion transporter | 267 |
| 18 | 462201 | 460643 | integral membrane protein | 1057 |
| 18 | 487971 | 483350 | ATP-dependent bile acid permease | 267 |
| 18 | 509079 | 514060 | ethanolamine phosphate transferase | 259 |
| 18 | 538803 | 536453 | transcription factor | 294 |
| 18 | 618965 | 617926 | histidine kinase response regulator sensor | 253 |
| 18 | 662138 | 665021 | ABC transporter | 202 |
| 19 | 36330 | 38772 | mannosyltransferase | 451 |
| 19 | 44962 | 45258 | short-chain dehydrogenase/reductase | 784 |
| 19 | 169271 | 167363 | MAP kinase | 153 |
| 19 | 172270 | 173374 | G protein complex α subunit | 354 |
| 19 | 196563 | 195631 | peptidyl-prolyl cis-trans isomerase | 249 |
| 19 | 219256 | 217310 | choline dehydrogenase | 922 |
| 19 | 381381 | 378112 | phospholipase | 373 |
| 19 | 450648 | 449807 | oligopeptide transporter | 1085 |
| 19 | 455190 | 454687 | MFS multidrug transporter | 511 |
| 19 | 502632 | 504088 | polyketide synthase | 40 |
| 19 | 530661 | 529821 | endoglucanase | 566 |
| 19 | 540198 | 538221 | Zn(II)(2)Cys(6) transcription factor | 734 |
| 20 | 24190 | 26297 | cutinase transcription factor | 1021 |
| 20 | 32447 | 34456 | peptide transporter | 1085 |
| 20 | 116411 | 117679 | phenylacetyl ligase | 508 |
| 20 | 155433 | 154666 | transcription factor | 273 |
| 20 | 158802 | 160686 | carboxypeptidase | 903 |
| 20 | 190666 | 184578 | histidine kinase response sensor | 562 |
| 20 | 226386 | 227738 | pathogenesis associated protein | 27 |
| 20 | 248693 | 249257 | cell pattern formation-associated protein | 776, 802 |
| 20 | 419715 | 422113 | ABC multidrug transporter | 1018 |
| 20 | 433052 | 430866 | ABC transporter | 310 |
| 20 | 459445 | 457757 | MFS multidrug transporter | 510 |
| 20 | 506170 | 503988 | two-component histidine protein kinase sensor | 562 |
| 20 | 515641 | 516958 | allantoicase | 854 |
| 21 | 17387 | 15356 | trichothecene efflux pump | 131 |
| 21 | 41214 | 40003 | histone deacetylase | 260 |
| 21 | 61781 | 63289 | polygalacturonase | 115 |
| 21 | 76198 | 79092 | kinesin | 465 |
| 21 | 110825 | 112862 | 4-coumarate ligase | 508 |
| 21 | 220197 | 218323 | ribonuclease Z | 444 |
| 21 | 231857 | 230017 | laccase-1 | 552 |
| 21 | 249467 | 244094 | acyl-CoA ligase | 293 |
| 21 | 317445 | 315898 | DUF1237 domain-containing protein | 785 |
| 21 | 414536 | 415372 | aliphatic nitrilase | 143 |
| 21 | 434512 | 433119 | transferase | 867 |
| 22 | 31162 | 30413 | kinesin heavy chain | 465 |
| 22 | 38270 | 43222 | high affinity phosphodiesterase | 856 |
| 22 | 162662 | 161350 | peroxiredoxin | 386 |
| 22 | 169937 | 168886 | Rho GTPase | 808 |
| 22 | 213068 | 211380 | ATP-dependent molecular chaperone | 463 |
| 22 | 219685 | 218567 | thiosulfate sulfurtransferase | 616 |
| 22 | 234346 | 233202 | serine/threonine protein phosphatase | 378 |
| 22 | 350511 | 349286 | mitogen-activated protein kinase | 736 |
| 22 | 356234 | 354055 | ATP-dependent RNA helicase | 423 |
| 22 | 378796 | 375553 | UDP-glucose:sterol glycosyltransferase | 243 |
| 22 | 381481 | 380118 | aspartic endopeptidase | 697 |
| 23 | 209745 | 207137 | aspartic-type endopeptidase | 68 |
| 23 | 239523 | 240314 | citrate synthase | 447 |
| 23 | 241150 | 242565 | mitochondrial DNA replication protein | 254 |
| 23 | 283821 | 286307 | mannosyl transferase | 454 |
| 23 | 292120 | 289868 | carnithine acetyl transferase | 120, 594 |
| 23 | 302396 | 299536 | mRNA binding protein | 882 |
| 23 | 382661 | 380811 | heavy metal translocating P-type ATPase | 244 |
| 24 | 50573 | 52401 | iron transport multicopper oxidase | 552 |
| 24 | 63279 | 65189 | choline dehydrogenase | 922 |
| 24 | 159905 | 159158 | lactoylglutathione lyase | 414 |
| 24 | 283495 | 283791 | choline dehydrogenase | 922 |
| 25 | 41239 | 38690 | plasma membrane calcium-transporting ATPase | 440 |
| 26 | 76184 | 74732 | endoglucanase | 566 |
| 26 | 106954 | 108870 | MFS multidrug transporter | 511 |
| 26 | 153073 | 149386 | ABC transporter | 867 |
| 26 | 160347 | 163338 | sodium P-type ATPase | 440 |
| 27 | 42716 | 37816 | ABC bile acid transporter | 267 |
| 27 | 54384 | 78441 | nonribosomal peptide synthase | 160 |
| 27 | 126776 | 128538 | serine carboxypeptidase | 903 |
| 28 | 36542 | 38248 | 26S protease regulatory subunit 4 | 226 |
| 28 | 67069 | 65994 | NAD dependent epimerase dehydratase | 1047 |
| 28 | 139069 | 139638 | MIPC synthase subunit | 364 |
| 28 | 184932 | 182591 | polyketide synthase | 255 |
| 28 | 189528 | 194457 | ABC multidrug transporter | 267 |
| 29 | 67346 | 68851 | acyltransferase | 167 |
| 29 | 141998 | 142886 | FAD-binding domain containing protein | 1046 |
| 32 | 6323 | 5368 | UDP transferase | 243 |
| 32 | 87344 | 85369 | O-methyl transferase | 1051 |
| 33 | 40632 | 42662 | catalase | 106 |
| 34 | 48371 | 47334 | N-acetylglucosamine-6-phosphate deacetylase | 207 |
| 34 | 65572 | 66157 | glucosamine-6-phsophate deaminase | 206, 221 |
| 40 | 5399 | 3752 | saccharopine dehydrogenase | 384 |
